# Confocal Raman Spectroscopic Characterization of Dermatopharmacokinetics Ex Vivo

**DOI:** 10.1021/acs.molpharmaceut.3c00755

**Published:** 2023-10-06

**Authors:** Panagiota Zarmpi, M. Alice Maciel Tabosa, Pauline Vitry, Annette L. Bunge, Natalie A. Belsey, Dimitrios Tsikritsis, Timothy J. Woodman, M. Begoña Delgado-Charro, Richard H. Guy

**Affiliations:** † Department of Life Sciences, University of Bath, Claverton Down, Bath BA2 7AY, U.K.; ‡ Department of Chemical & Biological Engineering, Colorado School of Mines, Golden, Colorado 80401, United States; § National Physical Laboratory, Teddington TW11 0LW, U.K.; ∥ School of Chemistry & Chemical Engineering, University of Surrey, Guildford GU2 7XH, U.K.

**Keywords:** Raman spectroscopy, skin uptake, skin clearance, skin penetration, topical bioavailability

## Abstract

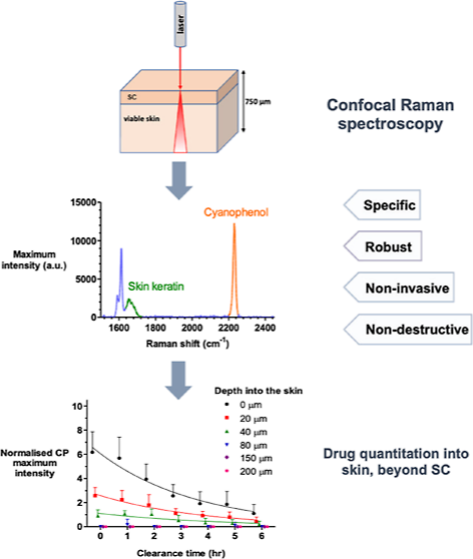

Confocal Raman spectroscopy is being assessed as a tool with which to quantify the rate and extent of drug uptake to and its clearance from target sites of action within the viable epidermis below the skin’s stratum corneum (SC) barrier. The objective of this research was to confirm that Raman can interrogate drug disposition within the living layers of the skin (where many topical drugs elicit their pharmacological effects) and to identify procedures by which Raman signal attenuation with increasing skin depth may be corrected and normalized so that metrics descriptive of topical bioavailability may be identified. It was first shown in experiments on skin cross-sections parallel to the skin surface that the amide I signal, originating primarily from keratin, was quite constant with depth into the skin and could be used to correct for signal attenuation when confocal Raman data were acquired in a “top-down” fashion. Then, using 4-cyanophenol (CP) as a model skin penetrant with a strong Raman-active C≡N functionality, a series of uptake and clearance experiments, performed as a function of time, demonstrated clearly that normalized spectroscopic data were able to detect the penetrant to at least 40–80 μm into the skin and to distinguish the disposition of CP from different vehicles. Metrics related to local bioavailability (and potentially bioequivalence) included areas under the normalized C≡N signal versus depth profiles and elimination rate constants deduced post-removal of the formulations. Finally, Raman measurements were made with an approved dermatological drug, crisaborole, for which delivery from a fully saturated formulation into the skin layers just below the SC was detectable.

## Introduction

To effectively measure the rate and extent at which a drug reaches its site of action requires the ability to interrogate and quantify the “local” uptake and clearance events.^1^ For orally administered drugs, this assessment is typically made using the blood, serum, or plasma as a “surrogate” compartment.^2^ For drugs, which are applied in topical formulations to treat dermatological disease, the pharmacological targets are most often located in the viable tissue layers (epidermis/dermis/appendages) beneath the skin’s formidable stratum corneum (SC) barrier and, therefore, the quantification in the systemic compartment is not generally considered suitable for the measurement of local bioavailability.^3^

Alternative approaches to resolve this challenge (as summarized in a recent publication^4^) are the subject of much recent research, and it has been hypothesized that Raman spectroscopy offers a non-invasive, accurate, and reproducible tool with which topical drug bioavailability may be characterized.^1,5−7^ Recent work has shown that the application of Raman spectroscopy can objectively compare different formulations in terms of their ability to deliver a chemical into the skin and that the results correlate well with data acquired—from the same tissue samples—with the well-studied (but labor-intensive) approach of SC sampling using adhesive tape-stripping.^4^

In addition to those mentioned already, other advantages of Raman spectroscopy include its non-destructive nature and specificity, enabling the spatial and temporal identification of a drug within the skin post-application of a topical formulation.^5,8,9^ The inelastic scattering of light by matter (Raman effect) is unique for specific molecular vibrations and offers a truly label-free technique.^6^ Furthermore, as the generated Raman intensity is linearly proportional to the chemical’s concentration in the medium of interest, direct quantification from the spectroscopic measurements is, in principle, possible.^10^ Finally, the evolution of Raman spectroscopy to Raman microscopy permits chemical imaging in a heterogeneous tissue and the investigation of mechanistic hypotheses to be addressed.^11^ From a regulatory perspective, Raman spectroscopy merits evaluation as an approach that may usefully complement other methods under consideration as surrogates for the determination of topical drug bioavailability and topical product bioequivalence.

However, while the use of Raman spectroscopy in confocal mode can probe beyond the SC and closer to the location of drug targets in the living skin beneath the SC, two important technical challenges must be surmounted to render the technique worthy of detailed examination. First, as Raman signals are known to be relatively weak, it must be shown that unambiguous detection of the drug in deeper layers of the skin is possible even though there may be appreciable background signal interference from the skin and/or other constituents of the applied formulation.^11^ Second, the confocal Raman approach necessarily suffers from signal attenuation, due to the absorption and scattering as data are collected from progressively deeper positions within the skin.^12^ The planar interface between regions of different refractive indices (air and the illuminated sample, in this case, the skin) introduces spherical aberration, especially when working with air objectives, decreasing the depth resolution and further contributing to signal intensity loss.^13,14^ It is therefore essential to identify and validate a suitable strategy to correct for signal loss appropriately. A generally reliable method to study and further correct the attenuation of signals with depth is to examine the material of interest (in this case skin) laterally. So far, this approach has been introduced to investigate the exact depth of focus when performing depth mapping using polymeric matrices but can serve as a tool to identify the appropriate reference metric (within the skin) with which to correct confocal Raman data.^15,16^

These objectives are addressed in this work in a series of experiments using porcine skin ex vivo, a widely accepted and well-validated representation of the human counterpart.^17^ Following on from previous studies,^7^ 4-cyanophenol (CP)—which has an intense Raman signal (C≡N vibration) at a frequency where skin and typical formulation excipients do not—has been selected as the model permeant. In addition to its favorable Raman characteristics, CP can be considered a “good” skin penetrant^18^ and therefore provides a suitable tool with which to examine the research questions posed about the feasibility of using this spectroscopic technique to assess local availability after topical application of a dermal drug product; in other words, if the approach does not work using CP, then its wider application to drugs with less optimal spectroscopic and percutaneous permeation properties will be a particularly challenging goal. The latter may be achievable, at least in some cases, as demonstrated in the final part of this research using crisaborole, an approved drug for the treatment of atopic dermatitis.^19^

## Materials and Methods

### Materials

CP, crisaborole, propylene glycol (PG), propylene carbonate, and all other solvents and reagents were obtained from Sigma-Aldrich (Dorset, UK). Fresh abdominal porcine skin from two animals was obtained from a tissue supplier and then dermatomed (Zimmer, Hudson, OH, USA) within 24 h of slaughter to a nominal thickness of 750 μm. Visually obvious hairs were carefully cut away with scissors, and the tissue was then stored at −20 °C until being thawed before use. All CP experiments were conducted with skin samples from one pig, while those for crisaborole used tissue from the second animal.

### Ex Vivo Measurements of Chemical Uptake into and Clearance from the Skin

CP disposition in the skin was evaluated following the application of three distinct formulations: (a) 170 mg mL^–1^ of CP in 50:50 v/v water/PG for 1, 2, and 6 h, (b) 17 mg mL^–1^ of CP in 90:10 v/v water/PG for 1 and 2 h, and (c) 42.5 mg mL^–1^ of CP in 50:50 v/v water/PG for 6 h. The first two formulations represented saturated CP solutions and the third was a 25% saturated solution (Maciel Tabosa et al., 2023). The formulations (300 μL) were applied under occlusion (Parafilm, Bemis Company, Inc., Neenah, USA) to porcine skin mounted in a Franz cell (PermeGear, Hellertown, PA), with a diffusion area of 2.01 cm^2^, thermostated at 32 °C. The receptor solution was pH 7.4 phosphate-buffered saline (PBS).

Crisaborole disposition in the skin was assessed in a similar experimental setup. The formulation examined was fully and half-saturated solutions in propylene carbonate (90 and 45 mg mL^–1^); the drug’s solubility in the vehicle was determined using the shake-flask method. The volume of each formulation applied, in this case for 24 h, was again 300 μL. The uptake time was selected to boost crisaborole uptake into the skin and therefore amplify the spectroscopic signals.

**Figure 1:**
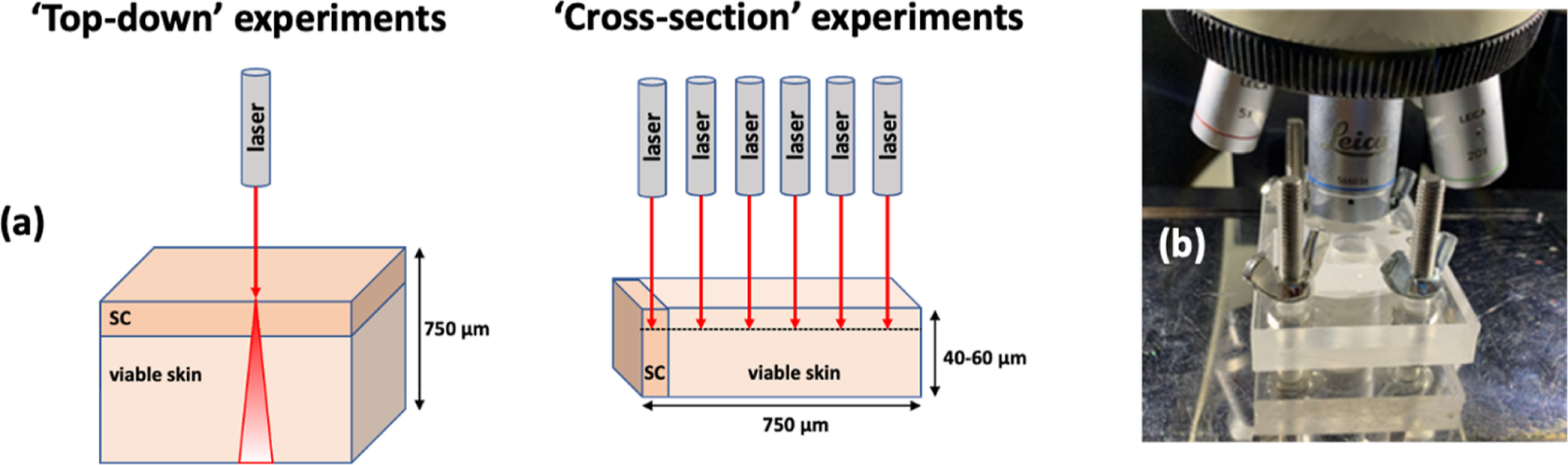
(a) Schematic of “top-down” and “cross-section” experiments. (b) Custom-built skin sample holder. SC = stratum corneum.

At the end of each experiment, the skin was cleaned with dry tissue and then sectioned into smaller pieces. Chemical uptake into the skin and its subsequent clearance therefrom was then assessed using Raman spectroscopy in parallel orientation to the skin surface in so-called “top-down” experiments (Figure 1a). A first section of skin was mounted in a simple, custom-built sample holder, adapted for use with the Raman microscope (Renishaw RM1000 Raman microscope running v1.2 WIRE software, Renishaw plc, Wotton-under-Edge, UK) that permitted tissue hydration to be maintained via a small PBS-filled well beneath the skin (Figure 1b). For CP, the uptake “top-down” profile was then acquired from the skin surface (0 μm) to a depth of 100 μm at 20 μm intervals. Amide I signals were acquired at the same time. Following a 6 h application of each formulation, sequential Raman measurements were recorded of CP clearance from the skin over the next 6 h at 1 h intervals. New samples were prepared for the clearance experiments; that is, different skin pieces were used from those which provided the 6 h uptake (which is also the 0 h clearance) measurement. In this case, “top-down” profiles were acquired at 0, 20, 40, 80, 150, and 200 μm below the skin surface. For crisaborole, the uptake profile was acquired from the skin surface to a depth of 50 μm at 5 μm intervals; however, no clearance data were obtained for this drug. Control measurements were also made on untreated skin, which had been placed on a Franz cell with 300 μL of pH 7.4 PBS for 1 h.

In the cross-section experiments (Figure 1a), skin sections were snap-frozen at −25 °C in dry ice and cryo-microtomed (Leica CM1850, Leica, Germany) to a thickness of 40–60 μm. Raman spectra were then acquired (as for the “top-down” measurements but with the skin oriented horizontally, rather than vertically, in the sample holder) from just below the cut surface of the sections beginning at the SC and then progressively to a depth of 200 μm in 20 μm intervals. All “top-down” and “cross-section” experiments were performed with *n* = 6 (i.e., six experiments using different pieces of skin from a single animal).

### Confocal Raman Spectroscopy Data Acquisition and Analysis

A 1200-line/mm grating providing a spectral resolution of 1 cm^–1^ was used with a diode laser operating at 785 nm. The Raman band (520 cm^–1^) of a silicon wafer was used as a calibration reference sample to correct the Raman shift. For CP, an exposure time of 10 s with 20 accumulations and a laser power of 50% were used with a long 50× working distance objective lens. For crisaborole, an exposure time of 10 s with 40 accumulations and a laser power at 100% was used with the same working distance objective to improve the signal intensity. No signs of sample damage were observed under any of the above experimental conditions. All spectra were acquired over the wavenumber range of 1500–2450 cm^–1^, and signals from the C≡N (2200 and 2260 cm^–1^) and amide I (C=O; 1600–1720 cm^–1^) stretching vibrations were recorded. The critical level for CP or crisaborole detection (*A*_c_) was defined in each individual spectrum, as described before,^7^ and any measurements below this value were replaced by zero.

To confirm that the “top-down” signal attenuation as a function of depth is similar for all frequencies (i.e., not uniquely that of the amide I), additional confocal Raman spectra from untreated skin were acquired using a protocol identical to that employed in the CP experiments described above. Spectra were again acquired, at 20 μm intervals, from the skin surface (0 μm) to a depth of 200 μm over the wavenumber ranges 1059–2083 and 2715–3447 cm^–1^, recording signals specifically from (a) 1380–1500 cm^–1^ (CH_2_ bending from skin lipids and proteins), (b) amide I as above, and (c) 2820–3000 cm^–1^ (primarily CH_3_ symmetric stretching from lipids). Measurements were taken in triplicate (i.e., three different skin pieces from one pig).

To calculate the exponential loss of measured signal from attenuation with depth, the natural log-transformed maximum intensities of the amide I signal from the skin confocal spectra were linearly regressed as a function of skin depth and assessed in terms of goodness-of-fit (*r*^2^) using GraphPad Prism 5 (ver. 9.3.1, San Diego, CA). The same approach was used to assess the signal attenuation of the CH_2_ and CH_3_ vibrational modes.

The confocal Raman profiles of the signals of interest are presented in terms of maximum intensity in arbitrary units (a.u.) or corrected maximum intensity (also in a.u.), as a function of depth (μm). CP or crisaborole normalized intensities (in dimensionless units) were calculated by dividing their respective maximum intensities by those of the amide I in each spectrum and plotted as a function of skin depth (μm) or clearance time (h). For the uptake data, the areas under the curve of the Raman normalized intensities versus depth profiles (AUC_*z*_) were determined using the trapezoidal rule. For the clearance data, the areas under the curve of the Raman normalized intensities versus clearance time profiles (AUC_*t*_) were found using the same method. The first-order elimination rate (*k*_e_) was calculated by performing a linear regression on the natural log-transformed average values of the normalized CP intensity versus clearance time data at each depth (where applicable), again using GraphPad Prism 5. For each formulation, the calculated slopes at each depth were compared, and a pooled value (i.e., shared slope based on global fitting^20^) was calculated when the individual results were not statistically different at a 95% confidence level.

### CP “Calibration Curve”

To explore whether a “calibration curve” might be generated to convert Raman spectroscopic signals into chemical concentrations, a model based on lyophilized porcine skin powder was developed. Abdominal skin was thawed at room temperature for 30 min. The skin was then cut into small pieces, dipped into liquid nitrogen for a few seconds, and then freeze-dried (−40 °C, 0.1 mbar pressure) for 72 h (Micro Modulyo-230 freeze drier, Thermo Fischer Scientific, Massachusetts, USA). Immediately thereafter, the dried pieces were crushed into a filamentous powder using a domestic coffee grinder (Braun Aromatic Coffee Grinder KSM2-WH, Amazon, UK) and stored in a desiccator at room temperature. Calibration curve standards were subsequently prepared in aluminum pans used for differential scanning calorimetry. Ten milligrams of the lyophilized pig skin powder was weighed into each pan and spiked with 23.3 μL of aqueous CP solutions at different concentrations; the volume of liquid used was chosen to reflect the normal (∼70% w/w) hydration level of viable skin tissue. To minimize evaporative loss, the samples were prepared immediately before acquiring Raman measurements (in triplicate) from the surface of the powder slurry for each of the calibration standards. The normalized CP intensities were then plotted as a function of CP concentration in the powder (in mM) assuming that the density of the suspension was 1 g mL^–1^.

## Results

### Correction for Signal Attenuation

**Figure 2:**
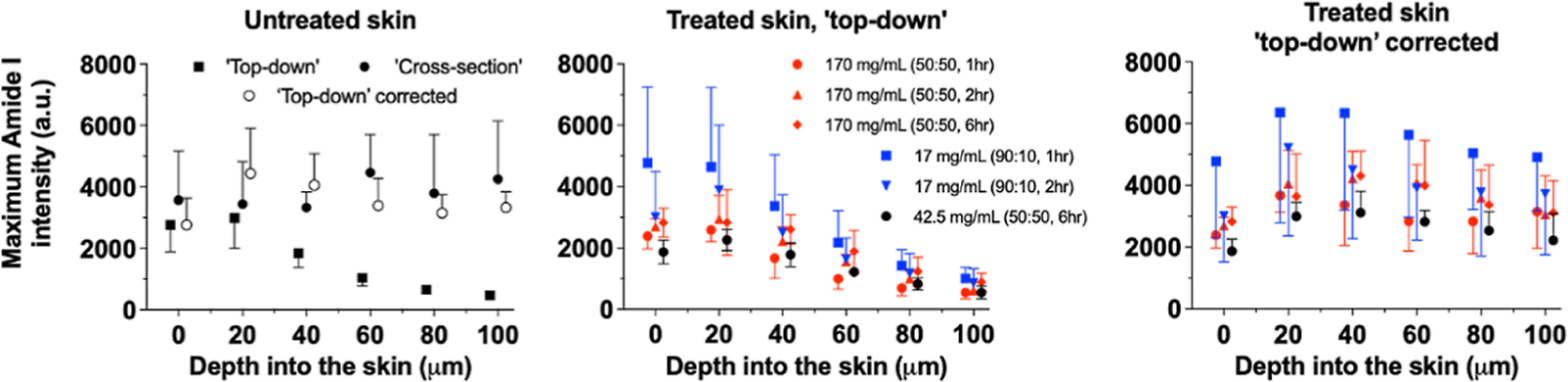
“Cross-section”, “top-down”, and corrected “top-down” maximum amide I signal intensities (for signal attenuation that increases with increasing depth into the skin, as described in the text) from untreated skin samples (left panel). The “top-down” and corrected for attenuation “top-down” data after application of different CP formulations for different times are shown in the central and right panels, respectively (some data points have been shifted on the *x*-axis to facilitate visualization). Data points represent the mean plus or minus the SD (from six different skin samples from a single pig).

The measured, maximum amide I signal intensities in the “cross-section” and “top-down” experiments from an untreated control are presented in the left panel of Figure 2. The “cross-section” data, although variable, consistently show that the amide I signal is substantially constant across the skin depth examined. In contrast, the “top-down” measurements decay monotonically with increasing depth into the skin, demonstrating unequivocally (when compared with the “cross-section” results) the attenuation of the signal due to absorption and scattering. It is noted that the latter does not appear to have been influenced in any systematic manner by the various applications of CP formulations to the skin (Figure 2, central panel).

The comparison of the “top-down” and “cross-section” amide I data provides a means by which to generally correct the former for the exponential signal attenuation observed with skin depth (consistent with previous reports in the literature^12,21^). In other words, the variation in the maximum amide I signal intensity (*I*_Amide_(*z*)) measured “top-down” as a function of depth (*z*) into the skin can be written
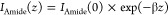
1where *I*_Amide_(0) is the unattenuated signal measured at the skin surface (*z* = 0) and β is the attenuation constant. For each individual experiment, *I*_Amide_(0) and β can be determined by linear regression of the “top-down” data to the natural log-transformed representation of eq 1, i.e.,
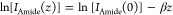
2

**Figure 3:**
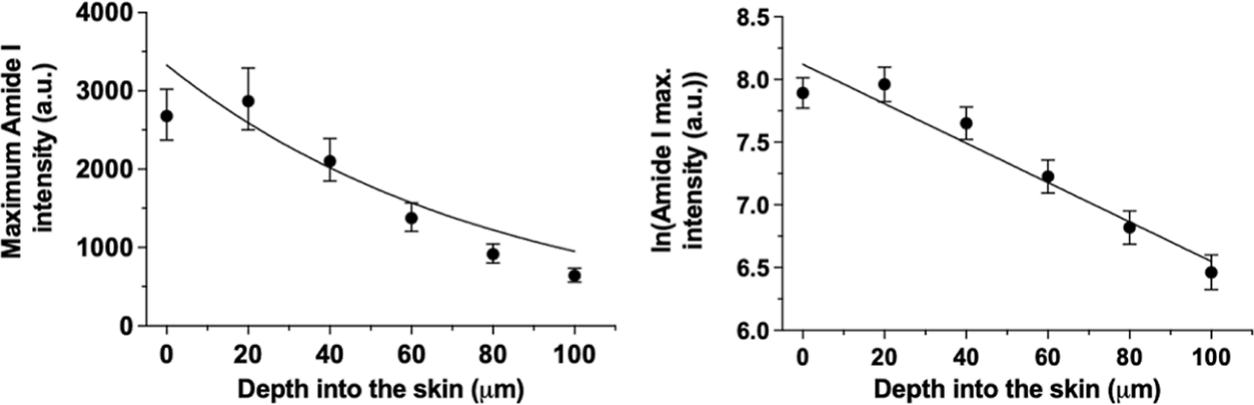
Combined, maximum amide I signal intensities (geometric means with their 95% confidence intervals) at 1655 cm^–1^ measured “top-down” as a function of skin depth (i.e., the data in the left and central panels of Figure 2, for the untreated and treated samples, respectively) and the natural logarithmic transformation of the data (means with their 95% confidence intervals) described by eq 2. Data points are calculated from experiments using 42 different skin samples from a single pig.

The “top-down” profiles shown in Figure 2 were corrected to their unattenuated values *I*_Amide,corr_(*z*) (i.e., those that would have been measured if this concentration were at the skin surface) using a rearrangement of eq 1
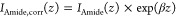
3and the β value determined in that same experiment. The *I*_Amide_(0) and β results derived from the linear regressions to eq 2 of the average ln(*I*_Amide_) versus depth values of the replicated experiments for each formulation and uptake time are summarized in Table 1. When all the data from the 42 experiments are combined (i.e., six replicates each for six combinations of formulation and time plus control, all measured in skin from a single pig), the values obtained for *I*_Amide_(0) and β (with their 95% confidence intervals) are 3364 a.u. (2402–4717 a.u.) and 0.0157 a.u./μm (0.0101–0.0213 a.u./μm), respectively, with *r*^2^ = 0.94 (Figure 3). No statistically significant differences between the values of β were observed (analysis of covariance,^20^*p* > 0.05; data not shown), but the *I*_Amide_(0) were different (analysis of covariance,^20^*p* < 0.0001; data not shown). Given that skin from a single pig was used in these experiments, it is reasonably assumed that the variability in the amide I intensities is not associated with differences in the amide I concentrations in the tissues but rather with variability in the Raman measurements, which had a similar effect on the CP signal (as discussed further below).

**Table 1:** Best-Fit Values (with 95% Confidence Intervals) of the Parameters Describing the Maximum Amide I Signal Attenuation (*I*_Amide_(0) and β) Determined by Linear Regression of the Average of the Natural Log-Transformed Amide I Measurements (6 Replicates) versus Depth to eq 2 in Experiments Involving Application of Three CP Formulations for 1, 2, or 6 h and an Untreated Control

experiment				
CP formulation	time (h)	*I*_Amide_(0) (a.u.)	10^2^ × β (a.u./μm)	*r*^2^
170 mg/mL in 50:50 v/v water/PG (fully saturated)	1	2876 (2052–4023)	1.75 (1.19–2.30)	0.95
	2	3481 (2310–5250)	1.60 (0.92–2.28)	0.91
	6	3361 (2373–4759)	1.25 (0.68–1.83)	0.90
17 mg/mL in 90:10 v/v water/PG (fully saturated)	1	4890 (3944–6057)	1.58 (1.23–1.94)	0.97
	2	3533 (2336–5340)	1.46 (0.78–2.14)	0.90
42.5 mg/mL in 50:50 v/v water/PG (25% saturated)	6	2492 (1575–3948)	1.40 (0.64–2.16)	0.87
untreated control	1	3378 (2343–4871)	1.97 (1.37–2.58)	0.95

**Figure 4:**
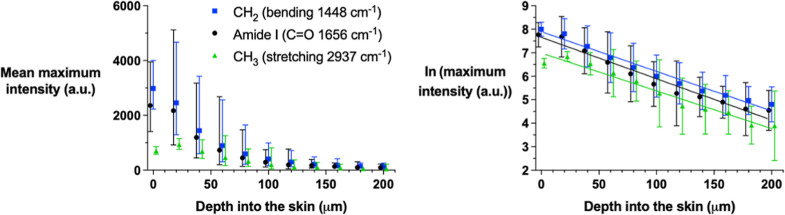
Maximum amide I, CH_2_ bending, and CH_3_ stretching signal intensities (geometric means and 95% confidence intervals) at 1655, 1448, and 2937 cm^–1^, respectively, measured “top-down” as a function of skin depth (left panel); the natural logarithmic transformations of the data (means and 95% confidence intervals) described by eq 2 are shown in the right panel. Data points (some of which have been shifted on the *x*-axis to facilitate visualization) are the means from three different skin samples from one pig.

The maximum amide I, CH_2_ bending, and CH_3_ stretching signal intensities measured in the further “top-down” experiments on untreated skin are presented in the left panel of Figure 4. All signals were attenuated with depth, independent of their *I*(0) values and the natural log-transformed data plotted as a function of depth were again linear (Figure 4, right panel). The β values with their 95% confidence intervals and *r*^2^ (derived from a linear regression to the average of the natural log-transformed data) are summarized in Table 2. Statistical analysis of these results revealed no difference between them (and provided a pooled β value of 0.0169 ± 0.0006 a.u./μm).

**Table 2:** Linear Regression Parameters (Best-Fit Values of β and the 95% Confidence Intervals for the Natural Log-Transformed Values of the Three Replicates Combined) for Measurements Using Untreated Skin, Describing the Amide I, CH_2_, and CH_3_ Signal Attenuation as a Function of Depth

	Amide I	CH_2_	CH_3_
10^2^ × β (a.u./μm)	1.76	1.69	1.61
95% confidence intervals (a.u./μm)	1.51–2.01	1.49–1.88	1.37–1.85
*r*^2^	0.97	0.98	0.96

### Confocal Raman Spectroscopic Measurement of CP Skin Uptake and Clearance

**Figure 5:**
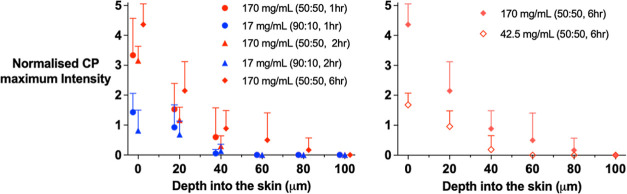
Normalized CP maximum intensity signal (eq 4) measured “top-down” as a function of skin depth following application of two fully saturated CP formulations in 50:50 or 90:10 v/v water/PG for 1, 2, or 6 h (left) and fully or 25% saturated CP formulations in 50:50 v/v water/PG for 6 h (right). Data points (some of which have been shifted on the *x*-axis to facilitate visualization) are the means (+SD) from six different skin samples from one pig.

Maximum C≡N signal intensities at ∼2230 cm^–1^ were acquired “top-down” after 1, 2, or 6 h applications of three different water/PG solutions of CP. As CP is a relatively good skin permeant, uptake times of 1 and 2 h were chosen for the two saturated formulations; the longer uptake time was used to better compare the 25% saturated solution in 50:50 v/v water/PG with the fully saturated one. In all cases, the raw signals decreased with increasing depth into the skin due not only to signal attenuation as discussed above but also because of the evolving concentration gradient as CP diffuses progressively into the skin.^22^ To reveal the actual profile of the permeant, it was therefore necessary to correct for the effect of signal attenuation in the data. This was achieved by assuming (further to the results in Figure 4 and Table 2) that the β value describing the attenuation of the amide I measurements in any experiment can also be used to correct the CP “top-down” signal intensity in the same way. In doing so, this effectively permits a “normalized” CP maximum intensity signal as a function of depth to be deduced directly from the ratio of “top-down” CP to amide I signals measured at each *z* in each experiment, i.e.,
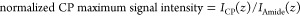
4The results described by eq 4 are summarized in Figure 5 (the raw CP intensities are shown in the Supporting Information, Figure S1) and highlight that the procedure for accounting for signal attenuation as a function of depth into the skin and for the normalization of the CP signal is straightforward. A second implicit assumption, however, is that the ratio *I*_CP_(*z*)/*I*_Amide_(*z*) is a relative measurement of the local skin concentration of CP, i.e., the mass of CP per mass (or volume) of skin. For this to be true, the amide I concentration (mass per mass of skin) in every piece of skin (i.e., the attenuated-corrected signal for amide I) must be constant. Although any specific trends in the data in Figure 2 are difficult to discern, the variability between the results is such that the validity of this assumption deserves more examination, taking into account, of course, the inherent (but not yet fully characterized) contribution of variability in the Raman spectroscopic measurements themselves. This issue is addressed in more detail in the Supporting Information.

As an additional metric with which to assess CP uptake into the skin from the Raman data, the areas under the normalized maximum signal versus skin depth profiles (AUC_*z*_) in Figure 5 were determined and are presented in Table 3.

**Table 3:** Areas under the Normalized CP Raman Signal Profiles (AUC_*z*_) as a Function of Skin Depth (Figure 5, Right Panel) Following Application of Three Water/PG Solutions for 1, 2, or 6 h (Mean ± SD, *n* = 6)

experiment		
CP formulation	time (h)	AUC_*z*_ (μm)
170 mg/mL in 50:50 v/v water/PG (fully saturated)	1	76 ± 48
	2	61 ± 18
	6	118 ± 47
17 mg/mL in 90:10 v/v water/PG (fully saturated)	1	34 ± 20
	2	25 ± 17
42.5 mg/mL in 50:50 v/v water/PG (25% saturated)	6	40 ± 19

**Figure 6:**
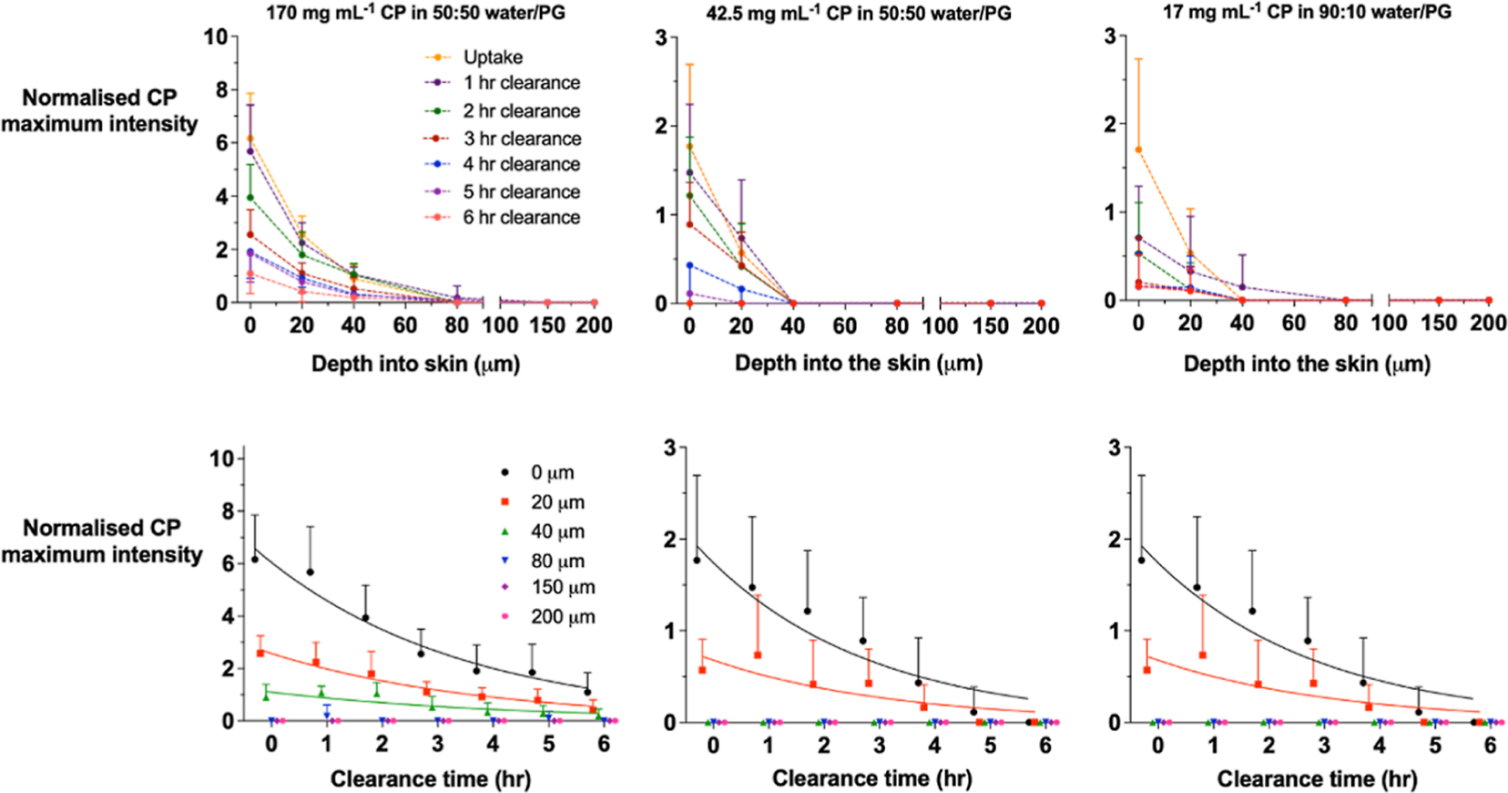
Upper panels: normalized CP maximum intensity signals as a function of skin depth at different clearance times, following the application of three CP formulations for 6 h. Dashed curves are simple connection lines between points. Lower panels: normalized CP maximum intensity signals as a function of clearance time at different depths into the skin, following application of the same three CP formulations again for 6 h. The lines are the best fits to an exponential decay function at each depth (for the cases when at least five non-zero average values were available). Some data points have been shifted on the *x*-axis to facilitate visualization. In both panels, the data points are the mean + SD (from six different pieces of skin from a single pig). Note that the *y*-axis scales are different for the 170 mg mL^–1^ CP formulation.

After application of the CP formulations for 6 h and cleaning of the skin surface, a series of clearance experiments were undertaken. “Top-down” CP Raman signals (and the corresponding amide I data) were acquired as before as a function of depth into the skin and as a function of clearance time, i.e., the time post-removal of the formulation. The normalized CP maximum signal intensities as a function of depth at different clearance times, or as a function of time at different skin depths, are presented in Figure 6; the raw amide I and CP intensities are shown in the Supporting Information, Figure S3. This set of profiles enables the calculation of the AUC_*t*_ (Table 4) and provides a metric relevant to an assessment of local bioavailability.

**Table 4:** Areas under the Normalized CP Raman Signal Profiles as a Function of Clearance Time (AUC_*t*_, Figure 6, lower panels) Following the Removal of the Three Water/PG Solutions after a 6 h Uptake (Mean ± SD, *n* = 6)

	AUC_*t*_ (h)		
skin depth (μm)	170 mg/mL CP in 50:50 v/v water/PG (fully saturated)	42.5 mg/mL CP in 50:50 v/v water/PG (25% saturated)	17 mg/mL CP in 90:10 v/v water/PG (fully saturated)
0	19.3 ± 4.4	5.0 ± 2.8	2.7 ± 2.8
20	8.6 ± 3.0	2.1 ± 1.8	1.2 ± 2.4
40	3.9 ± 1.8	0	0.2 ± 0.4
80	0.2 ± 0.4	0	0

### CP Calibration Curve

**Figure 7:**
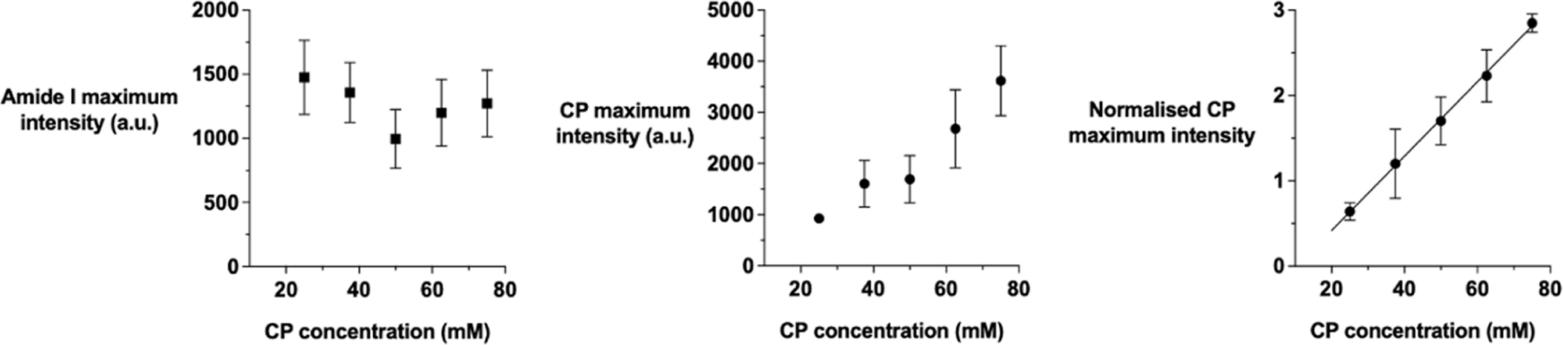
Amide I and CP maximum Raman signal intensities, together with the normalized CP to amide I ratio, as a function of CP concentration in the lyophilized and rehydrated pig skin powder model. Data points are the mean ± SD (*n* = 3) of the average value of three measurements of each standard. The regression line through the data in the right panel has a slope of 0.044 mM^–1^ (95% confidence interval is 0.041–0.046), with *r*^2^ = 0.999.

The results of the experiments attempting to establish a calibration curve for CP in a regenerated skin model are presented in Figure 7. The CP maximum and amide I Raman intensities and their ratios were acquired from the surface of the rehydrated skin powder as a function of the concentration of CP “doped” therein (with no attenuation). The normalized CP signals were linear with concentration over the range examined with *r*^2^ = 0.99. Since the amount of pig skin is the same in all model samples, the amide I intensities should be the same across the standards. Any variation in the amide I signal is then probably due to small differences in the precise location of the surface and/or spectroscopic variability (both of which would affect the amide I and CP maximum intensity equally). The normalization, in this case, provides an additional benefit as it should reduce the CP signal variation and (clearly) be more linear with CP concentration than the non-normalized data.

### Raman Assessment of Crisaborole Uptake into the Skin

**Figure 8:**
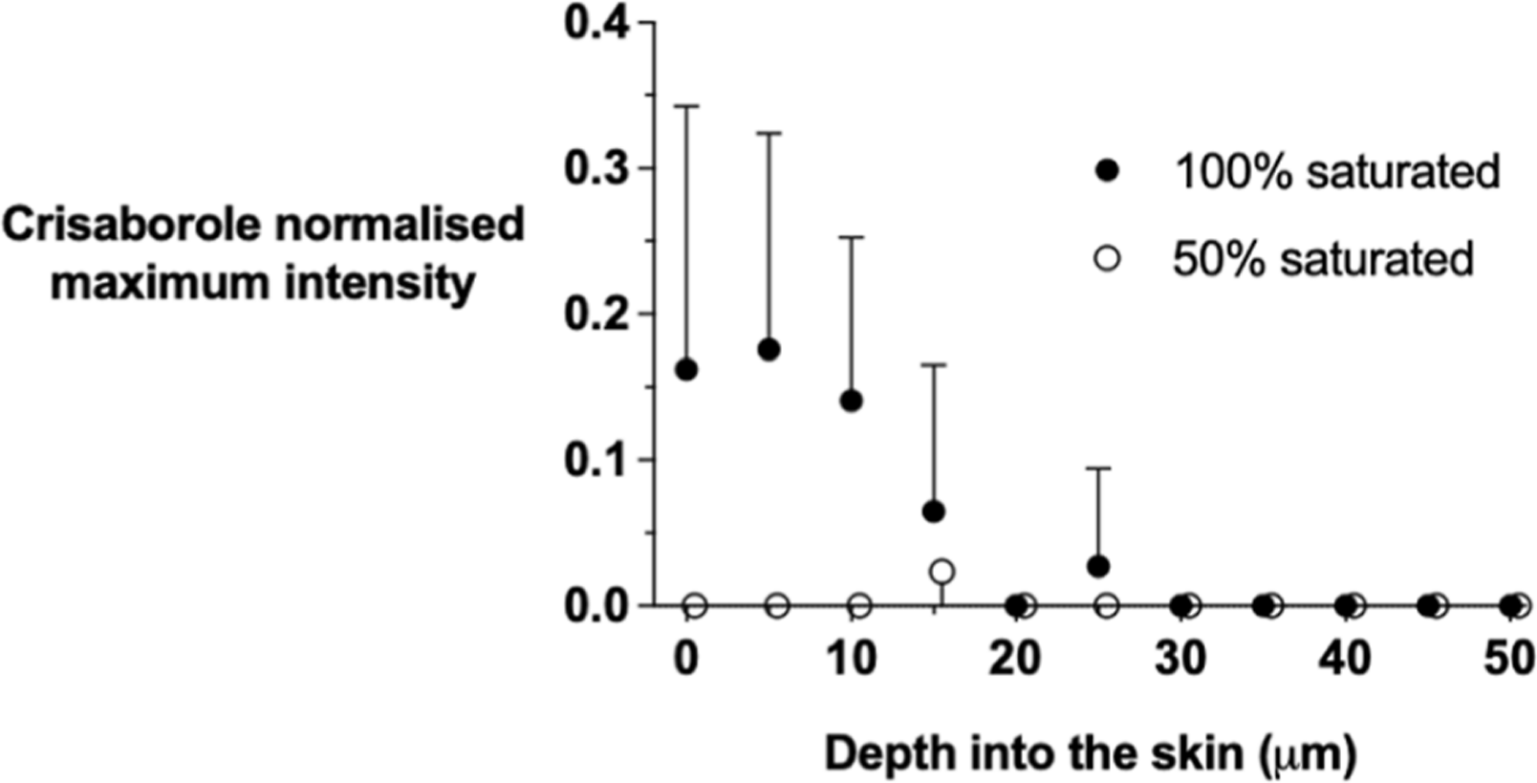
Normalized “top-down” maximum signal intensity of the crisaborole C≡N vibration at 2230 cm^–1^ as a function of skin depth following the application of fully saturated and half-saturated formulations in propylene carbonate for 24 h. Data points (some of which have been shifted on the *x*-axis to facilitate visualization) represent the mean plus or minus SD from *n* = 6 different pieces of skin from a single pig.

The uptake of crisaborole from fully and half-saturated solutions in propylene carbonate applied for 24 h was assessed in “top-down” Raman experiments that followed the method used for CP (Figure 8; the maximum amide I and crisaborole intensities are shown in the Supporting Information, Figure S4). Confocal Raman spectroscopy was able to detect crisaborole at skin depths beyond the SC (i.e., 10–20 μm) when the fully saturated vehicle was applied. Data from the half-saturated vehicle were (with one exception) below the critical level of detection at all depths.

## Discussion

The first objective of this study was to identify a strategy to correct for the progressive attenuation of the confocal Raman signal acquired as a function of increasing depth into the skin.^12,15^ Confocal Raman has been recognized as a relatively non-invasive tool with which to assess drug penetration into the skin from a topical formulation.^4,5,7,8^ As such, its application to quantify a drug’s local bioavailability requires that Raman signals detected are appropriately corrected to reflect the true concentration profile across the tissue.^12^

It was hypothesized that the amide I signal, originating primarily from the abundant keratin in the skin, should be relatively constant as a function of depth into the tissue. The “cross-section” experiments which permitted spectral examination of the SC and viable epidermis/upper dermis (Figure 1), confirmed that this was the case (Figure 2). A simple exponential decay equation was then proposed (eq 1, Figure 3) to correct the attenuated amide I data from “top-down” experiments and to validate the approach (Figure 2 and Table 1).

Differences in the calculated *I*_Amide_(0) values (from eq 2) from the CP uptake (and some control) experiments were detected. This is believed to be unrelated to any formulation effect as there was no correlation observed between *I*_Amide_(0) and the %PG present. An alternative hypothesis is that *I*_Amide_(0) reflects differences in the concentration of amide I in the samples and potentially undermines the assumptions inherent in the CP normalization approach described; however, this appears contradicted by the fact that data have shown quite consistently that when the raw amide I signal is noticeably higher than “normal” so is that from CP (Figure S1). This suggests that day-to-day (or even within-day) variation in the Raman measurements may be the major contributor to the differences in the observed amide I signals. Additional support for the assumption of consistency of the (depth-corrected) amide I signals across this investigation is that all skin samples used in the CP experiments were from the same animal (as were those for crisaborole but from a different pig).

Another approach to consider this question is to use a different normalization technique and to present the depth profile of CP in terms of its mass per mass of skin (rather than CP mass per mass of amide I). In other words, the maximum intensity signals for CP and amide I are assumed to represent their respective concentrations in the skin and the normalized metric becomes
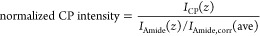
5where *I*_Amide,corr_(ave) represents the attenuated-corrected “average” amide I concentration in each skin sample, which is assumed, based on the attenuated-corrected results in Figure 2, to be invariant with depth and constant within each skin sample (but not necessarily between skin samples). This analysis produces profiles that are similar (but not identical) to the raw CP intensity versus depth profiles reported above (see Supporting Information, Figures S1 and S2) and confirms—at least for these CP experiments which used skin from a single source—that the analysis of the results is nonetheless valid. Clearly, further work (which will need to include interskin source variability) should be cognizant of the points raised here and the appropriate method(s) used to extract meaningful metrics from the data developed.

While variability in the absolute amide I signal was apparent, the within-skin consistency of the results from the cross-section experiments (and from the corrected “top-down” measurements) was good (Figure 2). It was noted that, in some of the “top-down” experiments, the mean amide I signal increased from 0 to 20 μm. This observation close to the sample surface has been previously reported in the literature.^13^ A likely explanation is imprecision in the exact position of the skin surface (i.e., where depth is set as 0 μm)^23,24^ due to the method's limited axial resolution. The axial resolution of the confocal Raman spectrometer is in fact on the order of several microns;^15^ that is, less than that achievable (typically, submicron) with more sophisticated Raman-based microspectroscopic approaches (coherent Raman scattering)^11^ or with laser scanning confocal fluorescence microscopy.^25^

Since amide I intensities are constant with skin depth, the described methodology could enable the correction (for attenuation) of all acquired signals, provided that the “loss” in intensity with depth is similar across different frequencies. To confirm this idea, additional depth profiles from untreated skin samples were acquired, and the intensities of Raman signals from other endogenous species (i.e., CH_2_ bending and CH_3_ stretching from skin lipids and proteins), in addition to amide I, were recorded (Figure 4). When the attenuation of these further Raman signals was fitted to eq 2, the derived values of β were very similar to each other and to that determined initially for amide I (Table 2) (analysis of covariance revealed no statistical differences between the slopes, *p* > 0.05). It, therefore, seems reasonable to assume that the signals of interest from CP and crisaborole will be attenuated as a function of depth in the same way and that their intensities can be corrected accordingly by the β found from the amide I data.

Consequently, the CP signals (C≡N vibration at ∼2230 cm^–1^) were normalized by the corresponding amide I values to generate a semiquantitative profile across the skin. The assumption that the attenuation of the amide I and CP signals is the same is further justified by the fact that the two measurements were always acquired at the same time and from the same skin “coordinates”. Having verified the rigor of the spectral data handling method used, it was then possible to show that CP was detectable below the SC in “top-down” Raman spectra (Figure 5) and that the normalization of the permeant’s intensities to those of amide I with eq 4 was justified. CP was detectable to skin depths of at least 40 μm for the formulations studied here and increasing the uptake time (e.g., to 6 h) or the degree of CP saturation in the 50:50 water/PG vehicle, permitted CP detection at depths around 80 μm. Clearly, this represents a significant improvement—in terms of CP quantitation deeper into the skin—than that afforded by tape-stripping, which is limited to sampling only the SC.^7,18^

The areas under these normalized intensity–depth profiles (AUC_*z*_) were determined for three CP formulations applied for 1, 2, or 6 h (Table 3). Assuming that the maximum intensities of the CP and amide I signals are not affected by the formulation (or that both are affected in the same way) and that the amide I concentration is the same in all samples, visual inspection of the normalized CP versus depth curves shows that the saturated 170 mg/mL formulation in 50:50 v/v water/PG delivered more permeant into the skin than the saturated 17 mg/mL solution in 90:10 v/v water/PG. That is, even though CP was at its maximum thermodynamic activity in both formulations (and should, in theory, have resulted in an equal driving force for skin uptake),^26^ it is clear that PG is not simply an “innocent bystander” and may well enhance CP solubility in the SC as the cosolvent itself permeates into the barrier.^27^ More consistent, however, was the comparison of CP uptake from the fully and 25% saturated solutions in 50:50 v/v water/PG. While the ratio of the AUC_*z*_ values (∼3.0) was less than the 4-fold predicted value, the confocal Raman measurements clearly showed that CP delivery from these two formulations was different. In terms of the impact of application time, from both the 170 mg/mL CP solution in 50:50 v/v water/PG and the 17 mg/mL solution in 90:10 v/v water/PG, CP uptake was insensitive; no statistically significant differences were found between the AUCs of different uptake times for the 50:50 (ANOVA) or the 90:10 (two-tailed *t*-test) v/v water/PG formulations. This finding is consistent with the known rapid penetration of CP across the skin and the speed with which a quasi-steady state concentration profile can be established^18,28^

Further “top-down” experiments were then performed to characterize the clearance of CP from the skin once the applied formulation had been removed. Figure 6 presents the results following a 6 h uptake of the three CP solutions studied and shows the normalized CP signals as a function of clearance time at different depths. Table 4 reports AUC_t_ under the normalized profiles which, as expected, decrease with increasing depth into the skin. Consistent with the uptake results discussed above, the AUC_*t*_ results for the saturated CP solution in 50:50 v/v water/PG are higher (at all skin depths) than those in 90:10 v/v water/PG; for depths between 0 and 40 μm, the ratio of AUC_t_ for the 170–17 mg/mL formulations is between 7 and 20. Beyond 40 μm, meaningful calculations of the ratio cannot be made due to the Raman signals falling below the critical level of detection.^7,29^ Also, in agreement with the uptake data, AUC_*z*_ values for the fully saturated 50:50 v/v water/PG CP solution are uniformly higher than those for the 25% saturated formulation; in this case, the ratios (down to 20 μm) fall around 4, consistent with the saturation levels of the applied CP solution.

The normalized CP clearance versus time data in Figure 6 were also fitted to a first-order decay, and an elimination rate constant (*k*_e_) was generated by a linear fit to the natural log-transformed data; the values of *k*_e_ are collected (except when insufficient data above the limit of quantitation were available) in Table 5. For the three formulations, the pooled averages of *k*_e_ for each of the three formulations from the available depths combined [0.30 (±0.02) h^–1^, 0.43 (±0.08) h^–1^, and 0.32 (±0.06) h^–1^ for the 170, 42.5, and 17 mg mL^–1^ solutions, respectively] were not significantly different.

**Table 5:** CP Elimination Rate Constants from the Skin (*k*_e_) as a Function of Clearance Time (Figure 6, Lower Panels) Following the Removal of Three Water/PG Solutions after a 6 h Uptake (*n* = 6)

	*k*_e_ (h^–1^) [(95% confidence interval); *r*^2^]		
skin depth (μm)	170 mg/mL CP in 50:50 v/v water/PG 100% saturated	42.5 mg/mL CP in 50:50 v/v water/PG 25% saturated	17 mg/mL CP in 90:10 v/v water/PG 100% saturated
0	0.29 [(0.35–0.23); 0.97]	0.51 [(0.81–0.20); 0.84]	0.40 [(0.59–0.21); 0.85]
20	0.29 [(0.36–0.23); 0.96]	0.31 [(0.66–0.05); 0.71]	0.24 [(0.43–0.05); 0.68]
40	0.30 [(0.43–0.18); 0.87]		

The results presented so far support the idea that the confocal Raman spectroscopy can generate—once corrected for signal attenuation by normalizing with respect to (for example) a constant (with depth and between skin samples) amide I intensity—suitable metrics with which the rate and extent of drug uptake into (and its subsequent clearance from) the skin can be deduced. Clearly, though, the metrics are relative rather than absolute quantities, with their normalized C≡N signal intensities from CP, while seemingly proportional to local concentrations (provided that amide I concentrations are similar within samples and that instrumental sensitivity between samples is the same), lacking any means by which they can be converted into absolute values. An initial attempt was made, therefore, to examine whether a rehydrated lyophilized skin powder model might be used to generate a “calibration curve” to translate spectral signals into concentrations. The results of this effort are summarized in Figure 7 and show that it is possible to generate a linear relationship between the normalized maximum intensity CP signal with CP concentration in the skin powder model. However, an independent validation of the approach has proved difficult to realize and remains a challenge. A particularly awkward issue is that, although the model employed is essentially homogeneous, intact skin is not, with the SC having distinctly different physicochemical properties to those of the underlying tissue; it is unlikely, therefore, that the same “calibration curve” will be applicable to these quite different skin compartments. There is also the question of sensitivity in that (as has been observed repeatedly over the years), the concentration of a skin penetrant in the SC is substantially greater than that achieved in the underlying viable tissue. Further work is warranted to address these questions in greater detail.

In a final series of experiments, the Raman-assessed skin uptake of an approved topical drug crisaborole was briefly investigated. While CP, with its high skin permeability and favorable Raman spectral features, is a useful model, it differs from most topical drugs in current use that are typically of higher molecular weight, more lipophilic, and significantly less permeable across the skin.^30^ Crisaborole was chosen as the first “real” drug for confocal Raman assessment because, like CP, the molecule also possesses a C≡N functional group and offers an advantageous Raman signal for easy detection. Fully and 50% saturated crisaborole formulations in propylene carbonate were considered. “Top-down” confocal Raman experiments were performed after a 24 h application of the crisaborole solutions; the extended exposure time was chosen to permit a greater uptake of the more slowly penetrating drug (as compared to CP). The results are shown in Figure 8 and demonstrate that the crisaborole can be detected at skin depths beyond the SC. However, only the measurements from the fully saturated formulation at depths up to 25 μm were above the critical level of detection. This indicates that method development—including, inter alia, increased data accumulation times, laser power, and spectral unmixing techniques (to better differentiate drug signals from those coming from endogenous skin species)—will be essential for Raman signals to be unambiguously detected in the “living” skin for drugs that penetrate the barrier slowly.

## Conclusions

Further to a recent publication^7^ that demonstrated the utility of vibrational spectroscopy to non-invasively assess the uptake of a topically applied chemical into the SC, the research presented here aims to show that confocal Raman spectroscopy, in particular, can follow a molecule’s disposition below the SC barrier and deeper into the skin. The results discussed above show that unambiguous Raman spectroscopic analysis of a model penetrant (CP) in the skin can be achieved to depths on the order of approximately 100 μm and that a robust approach to correct for signal attenuation as a function of the increasing depth of measurement has been validated. The normalization of the Raman signals then permits metrics ,describing chemical uptake into the skin and its clearance therefrom, to be derived from the spectroscopic profiles acquired as functions of both time and depth into the skin. These parameters relate directly, of course, to the local bioavailability of the molecule in that part of the skin where many topical drugs elicit their therapeutic effects. The validation of the approach was then demonstrated using crisaborole, an approved drug for the treatment of atopic dermatitis.

Although promising overall, further work is indicated in response to the data presented here. First, it is important to confirm the disposition data with measurements that permit an improved depth resolution compared with that of the confocal Raman apparatus used in this work. Coherent Raman scattering microscopies are more sophisticated tools, allowing depth resolution to the order of 1 μm or less. Second, because of the associated label-free and high-quality imaging capabilities of these methods, mechanistic questions concerning formulation metamorphosis and pathways of penetration, for example, can be investigated directly.^8^ Third, it is essential to identify the range of compounds to which Raman tools may be usefully applied; that is, to demonstrate that the methodology can also enable drugs with less favorable Raman characteristics than CP to be studied as well. Fourth, the development of the method to generate data that reflect drug disposition profiles in real rather than relative concentrations (or estimations thereof) is an important objective. Finally, it is clear that Raman spectroscopy is not a panacea for tracking of all topical drugs. As articulated above, the identification of a suitable spectroscopic signal from the drug, separable from background interference from the skin or formulation excipients, is an essential criterion for the application of the approach; the sensitivity limit of Raman also means that drugs that penetrate the skin more easily have a greater chance of being detectable within the viable tissue. Molecules like crisaborole, tazarotene (a third-generation retinoid), and members of the family of Janus kinase (JAK) inhibitors, with C≡C or C≡N substituents, are potentially attractive candidates for investigation, but other drugs with distinct Raman signals in the “fingerprint” region of the spectrum (such as metronidazole^31^) are also worthy of consideration.

## Supporting Information Available

Measured and normalized maximum CP signal intensities; Raman spectroscopy-assessed amide I and CP maximum signal intensities; and amide I and crisaborole maximum signal intensity of two saturated solutions (PDF)
