# Supplementary material for: Confocal Raman Spectroscopic Characterization of Dermatopharmacokinetics Ex Vivo
Source: Mol Pharm. 2023 Oct 6;20(11):5910–20. doi: 10.1021/acs.molpharmaceut.3c00755 (PMC10630943; doi:10.1021/acs.molpharmaceut.3c00755)
Supplement: Supplementary file 1 — mp3c00755_si_001.pdf [file mp3c00755_si_001.pdf]

Supplementary Information

Confocal Raman spectroscopic characterisation of dermatopharmacokinetics *ex vivo*

Panagiota Zarmpi[1‡], M. Alice Maciel Tabosa[1‡], Pauline Vitry[1], Annette L. Bunge[2], Natalie A. Belsey[3,4], Dimitrios Tsikritsis[3], Timothy J. Woodman[1], M. Begoña Delgado-Charro[1], Richard H. Guy[1*]

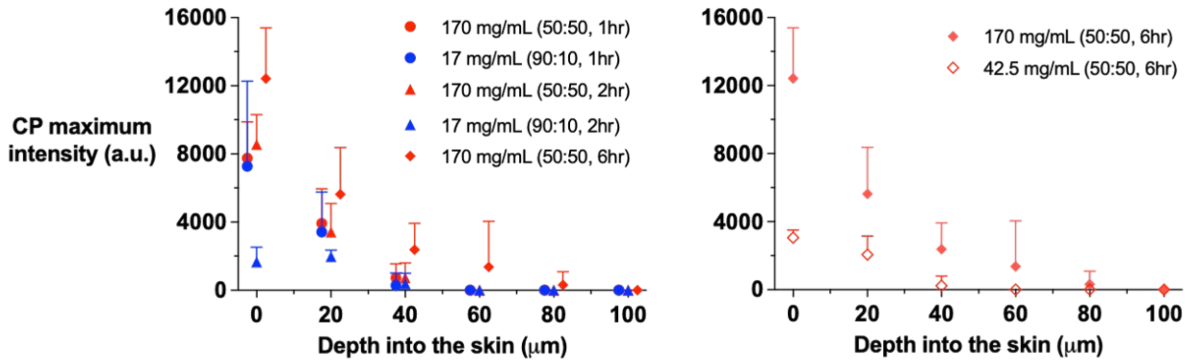

***Figure S1**: Maximum CP signal intensity measured 'top-down' as a function of skin depth following application of two fully saturated CP formulations in 50:50 or 90:10 v/v water/PG for 1, 2 or 6 hours (left), and fully or 25% saturated CP formulations in 50:50 v/v water/PG for 6 hours (right). Data points (some of which have been shifted on the x-axis to facilitate visualisation) are the means (+ SD) from 6 different skin samples from one pig.*

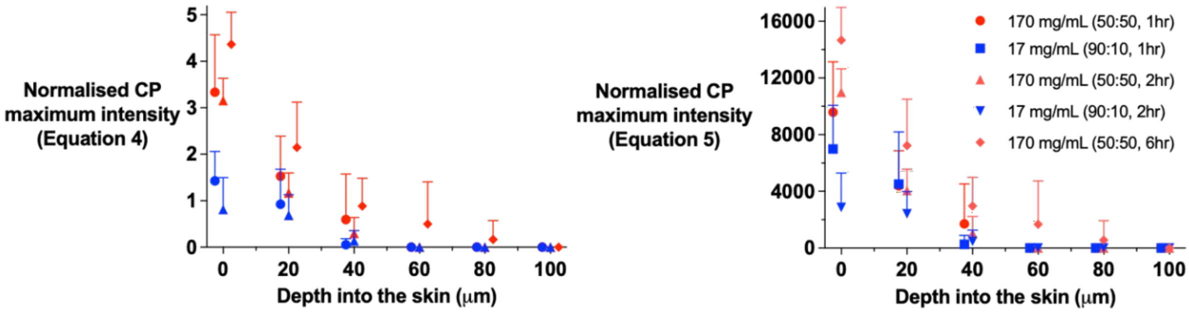

***Figure S2**: Normalised CP maximum intensity signal profiles based on Equation 4 (left) and Equation 5 (right); data were acquired 'top-down' as a function of skin depth following application of two fully saturated CP formulations in 50:50 or 90:10 v/v water/PG for 1, 2 or 6 hours. Data points (some of which have been shifted on the x-axis to facilitate visualisation) are the means (+ SD) from 6 different skin samples from one pig.*

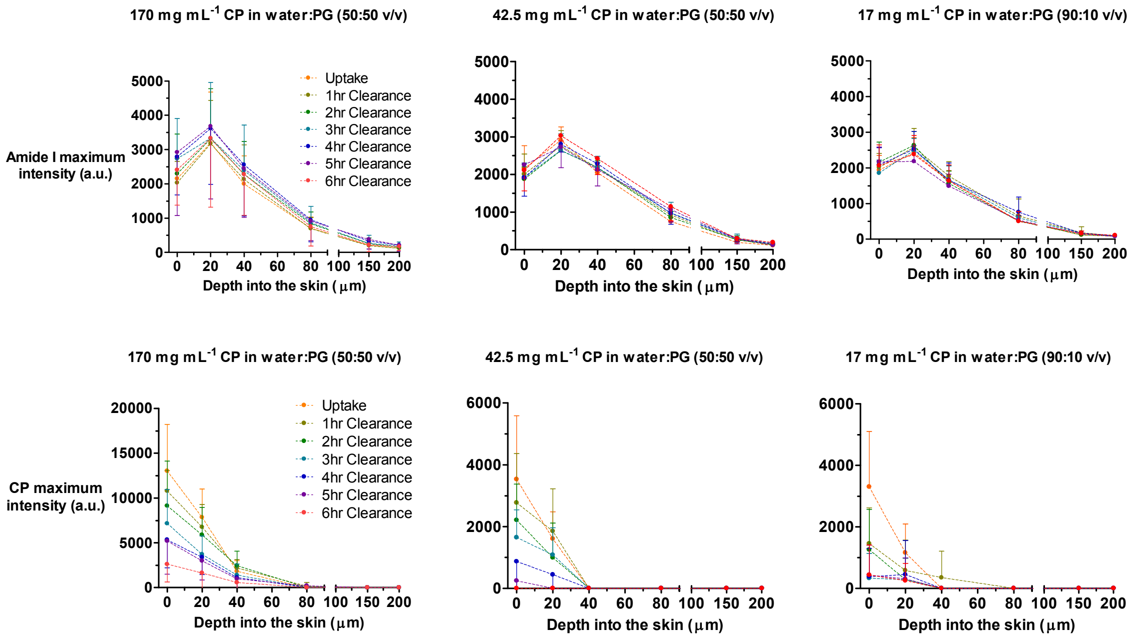

Figure S3: *Raman spectroscopy-assessed Amide I (top panels) and CP (bottom panels) maximum signal intensities measured 'top-down' as a function of skin depth following application of the three formulations indicated for 1, 2 or 6 hours. Data points represent the mean ± SD (n = 6 different pieces of skin from a single pig). Note the y-axis scales are different for the 170 mg mL^{-1} CP intensities.*

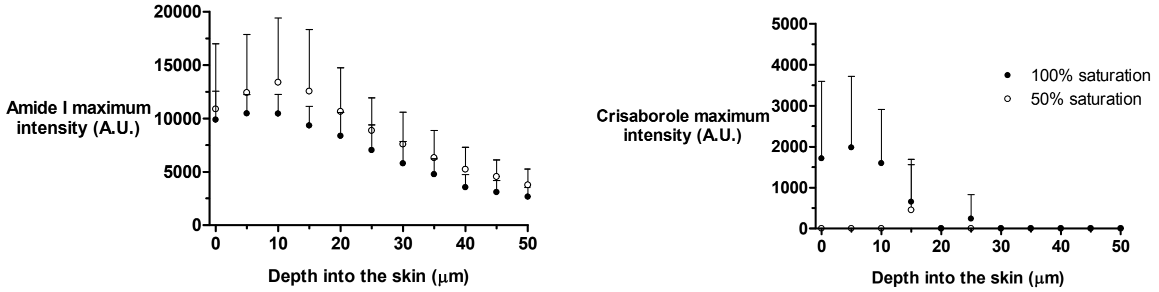

Figure S4: *Raman spectroscopy-assessed Amide I (left) and crisaborole (right) maximum signal intensity measured 'top-down' as a function of skin depth following application of the 100% and 50% saturated solution in propylene carbonate for 24 hours. Data points represent the mean + SD (n = 6 different pieces of skin from a single pig).*
